# 

**Published:** 1911-03-18

**Authors:** 


THE MODERN MEDICAL AND INSTITUTIONAL JOURNAL.

Telegraphic Address: Ospedale, London.

Telephone Number: 2734 Gerrard.

**NEW SERIES. No. 215, Vol. VIII. No. 1287, Vol. XLIX.** SATURDAY, MARCH 18TH, 1911. **PRICE THREEPENCE**

